# Tubular scaffold with microchannels and an H‐shaped lumen loaded with bone marrow stromal cells promotes neuroregeneration and inhibits apoptosis after spinal cord injury

**DOI:** 10.1002/term.2996

**Published:** 2020-01-29

**Authors:** Xue Chen, Jian Wu, Rongcheng Sun, Yahong Zhao, Yi Li, Jingying Pan, Ying Chen, Xiaodong Wang

**Affiliations:** ^1^ Department of Histology and Embryology, Medical College Nantong University Nantong Jiangsu China; ^2^ Wuxi Medical College Jiangnan University Wuxi Jiangsu China; ^3^ Jiangsu Key Laboratory of Neuroregeneration Nantong University Nantong Jiangsu China; ^4^ Key Laboratory for Neuroregeneration of Ministry of Education and Co‐innovation Center for Neuroregeneration of Jiangsu Province Nantong University Nantong Jiangsu China

**Keywords:** antiapoptosis, axonal regeneration, mesenchymal stem cell, scaffold architecture, spinal cord injury

## Abstract

As a result of its complex histological structure, regeneration patterns of grey and white matter are quite different in the spinal cord. Therefore, tissue engineering scaffolds for repairing spinal cord injury must be able to adapt to varying neural regeneration patterns. The aim of the present study was to improve a previously reported spinal cord‐mimicking partition‐type scaffold by adding microchannels on a single tubular wall along its longitudinal axis, thus integrating the two architectures of a single H‐shaped central tube and many microchannels. Next, the integrated scaffold was loaded with bone marrow stromal cells (BMSCs) and transplanted to bridge the 5‐mm defect of a complete transverse lesion in the thoracic spinal cord of rats. Subsequently, effects on nerve regeneration, locomotion function recovery, and early neuroprotection were observed. After 1 year of repair, the integrated scaffold could guide the regeneration of axons appearing in the debris of degraded microchannels, especially serotonin receptor 1A receptor‐positive axonal tracts, which were relatively orderly arranged. Moreover, a network of nerve fibres was present, and a few BMSCs expressed neuronal markers in tubular lumens. Functionally, electrophysiological and locomotor functions of rats were partially recovered. In addition, we found that BMSCs could protect neurons and oligodendrocytes from apoptosis during the early stage of implantation. Taken together, our results demonstrate the potential of this novel integrated scaffold loaded with BMSCs to promote spinal cord regeneration through mechanical guidance and neuroprotective mechanisms.

## 1 INTRODUCTION

Traumatic spinal cord injury (SCI) induces necrosis/apoptosis of neurons and oligodendrocytes, demyelination or axon degeneration, astrogliosis, and glial scar formation. Eventually, these processes result in the loss of sensation, paralysis, loss of bladder and bowel control, and sexual dysfunction (Chhabra & Sarda, 2017). In the past few decades, several approaches have been proposed to develop reasonable treatments for SCI repair, such as biomolecule‐ and cell‐based therapies (Antonic et al., 2013; Assinck, Duncan, Hilton, Plemel, & Tetzlaff, 2017; Ramer, Ramer, & Bradbury, 2014). However, tissue engineering may be a more effective approach for SCI repair, as it integrates strategies involving biomolecules, cells, and biomaterials (Führmann, Anandakumaran, & Shoichet, 2017; Ramer et al., 2014; Tuinstra et al., 2012). Biomaterials for nervous tissue repair are used as a bridge for axonal extension and scaffold for attachment of biomolecules and/or seed cells, especially for axon guidance. Thus, architecture and chemical properties of biomaterial‐based scaffolds are equally important.

Various types of scaffold architectures, such as single tube (ST), multiple channels, numerous microchannels, and cylindrical sponges, have been reported to guide the extension of regenerative axons (Bellamkonda, 2006; Bryant, Cuy, Hauch, & Ratner, 2007; Günther, Weidner, Müller, & Blesch, 2015; Madigan, McMahon, O'Brien, Yaszemski, & Windebank, 2009). However, the histological structure of the spinal cord is completely different from that of peripheral nerves, with the former being more complex. Spinal cord grey matter primarily contains neuronal somata and neural networks, whereas white matter is composed of linear axonal tracts; thus, these two nerve fibre pathways are very different. Regeneration of grey matter requires a larger space to allow cell migration and the formation of horizontal neural networks, whereas linear microchannels may be more suitable for axonal extension in white matter (Wong et al., 2008). In addition, different micro‐environments are required for growth and regeneration between white and grey matter of the spinal cord, and white matter contains primarily sensory or motor axons, respectively (Jones,Oudega, Bunge, & Tuszynski, 2001). For example, motor neurons containing TrkB and TrkC receptors are sensitive to brain‐derived neurotrophic factor (BDNF) or neurotrophin‐3, whereas sensory neurons, which contain TrkA receptors, are sensitive to nerve growth factor (Huang & Reichardt, 2003). As such, when applying tissue engineering for SCI, different active ingredients, such as a combination of several kinds of seed cells and/or cytokines, can be carried in different regions of scaffolds to achieve precision repair. Considering the above factors, if the morphology of scaffolds were similar to the histological structure of the spinal cord, in which the white and grey matter are divided into different regenerative regions, the repair effect would likely be further improved.

We previously reported a scaffold that simulated partitioned regeneration of spinal cord white and grey matter. In combination with neurotrophin‐3, this original scaffold provided obvious advantages for axon regeneration and functional recovery compared with an ST (Wang et al., 2013). However, the white matter region of the scaffold had only four channels on the dorsal side, which was insufficient to allow axonal tracts to pass through. Moreover, the effect of cytokines was temporary. Would it be better if the scaffolds contained seed cells (Willert h & Sakiyama‐Elbert, 2008). Because of the many advantages of bone marrow stromal cells (BMSCs), they have recently become widely used in cell therapy research and applications (Golpanian, Wolf,Hatzistergos, & Hare, 2016; Loebel & Burdick, 2018), including the exploration of treatments for central nervous system diseases with autologous transplantation or genetically modified BMSCs (Kandalam et al., 2017; Ritfeld et al., 2015). After transplantation of BMSCs into the central nervous system, there are five main mechanisms that promote the recovery of nerve function: neuroprotection, immune regulation, axon sprouting and/or regeneration, myelin regeneration, and neuronal relay formation (Assinck et al., 2017).

As such, the repair effect on SCI should be enhanced by both the improvement of scaffold architecture and loading with BMSCs. In this study, we further improved a spinal cord‐mimicking partition‐type scaffold by adding many microchannels along the longitudinal axis of the scaffold wall. This new scaffold architecture, which is equivalent to combining a single H‐shaped central lumen with many surrounding microchannels on the tubular wall, was designed to mimic the spinal cord architecture. After loading with green fluorescent protein‐labelled BMSCs (GFP‐BMSCs), scaffolds were utilized to bridge a 5‐mm‐long defect in rat thoracic spinal cords. The effects of repair were assessed over the course of 1 year using behavioural, electrophysiological, and histological methods. In addition, apoptosis of neurons and glial cells was observed to examine the neuroprotective role of BMSCs.

## 2 MATERIALS AND METHODS

### 2.1 Scaffold fabrication

ST and spinal cord‐mimicking partition‐type scaffolds were prepared as previously described (Wang et al., 2013). Briefly, chitosan (Nantong Xincheng Biochemical Company, Jiangsu, China) with an average molecular weight of 2.2 × 10^4^ D and deacetylation degree of 92.3% was dissolved in 2% acetic acid (Shanghai Ling Feng Chemical Reagents Company, Shanghai, China) at a ratio of 1 g per 20 ml, followed by addition of the same weight of chitin (Nantong Xincheng Biochemical Company). The gel mixture was injected into a −20°C stainless steel casting mould for 2.5 hr. After removal of the outer part of the mould, scaffolds with mould core were immersed in 10% NaOH (Shanghai Ling Feng Chemical Reagents Company) for 2 hr. Following rinsing with purified water (Milli‐Q®, Millipore, France) to a neutral pH, scaffolds were frozen and dried at −40°C and 0.02 Pa for 1 hr using a freeze dryer (Labconco, Kansas City, MO, USA). After removal of the mould core, scaffolds cast from different moulds were formed into ST with “O” shape lumen (Figure S1A,C) and spinal cord‐mimicking partition‐type scaffolds with “H” shape central lumen, respectively. Next, many microchannels were created by 250‐μm‐diameter acupuncture needles along the longitudinal axis of the latter scaffold on the tubular wall, which became an integrated scaffold of two relatively independent regeneration regions with a single H‐shaped central lumen for grey matter and many microchannels for white matter (ST/MC; Figure S1B,D). Before use, both types of scaffolds were disinfected with 20‐kGy 60Co radiation for 12 hr and then immersed in sterile saline for 30 min.

### 2.2 Cell preparation

OriCell™ Sprague Dawley rat GFP‐BMSCs (Cyagen Biosciences Inc., Shenzhen, China) used in this work are described in detail in Supporting Information. Passage 2 cells were employed for transplantation.

### 2.3 Animals and surgery

All experimental procedures and animal care were by the guidelines of the Institutional Animal Care and Use Committee of Nantong University and approved ethically by the Administration Committee of Experimental Animals of Jiangsu Province, China.

For surgeries, 129 adult female Sprague Dawley rats (200–220 g, purchased from the experimental animal centre of Nantong University) were anaesthetized intramuscularly (2.5 ml/kg) with a mixture of ketamine (62.50 mg/kg), xylazine (3.18 mg/kg), and acepromazine (0.63 mg/kg). A T8–T10 laminectomy was performed, and the dura was carefully opened using a longitudinal incision. The spinal cord was transected at approximately 4.5 mm from the level of T8 towards the caudal end and removed completely, thus leaving a defect approximately 5 mm long following retraction of the rostral and caudal ends. Motor evoked potentials (MEPs) were recorded at zero to ensure complete transection of the spinal cord. Rats were then randomly divided into six groups: ST (*n* = 15), ST/MC (*n* = 27), ST with GFP‐BMSCs (ST + BMSCs, *n* = 15), ST/MC with GFP‐BMSCs (ST/MC + BMSCs, *n* = 27), SCI without graft as a negative control (SCI, *n* = 12), and laminectomy only (sham, *n* = 21). In ST and ST/MC groups, 5‐mm scaffolds were used to bridge the spinal cord defect, followed by injection with 10 μl of phosphate‐buffered saline (PBS) into the lumen. In ST + BMSCs and ST/MC + BMSCs groups, scaffolds were implanted to bridge the gap as above, followed by injection with 10 μl of BMSC suspension (10^6^ cells/ml in PBS) into the lumen. In addition, 10 μl of a pan‐caspase inhibitor, z‐VAD‐fmk (Enzo Life Sciences, Farmingdale, NY), was injected into ST/MC as a control group (*n* = 12) for the apoptosis assay. Finally, the muscle and skin were stitched together layer by layer. Postoperatively, buprenorphine‐HCl (0.03–0.05 mg/kg) was subcutaneously administered twice a day for 2 days to relieve pain. Animals were then treated with penicillin (10^5^ U/kg) for 7 days as needed, and the bladder was manually emptied twice a day until the reflex recovered.

### 2.4 Functional tests

To ensure assessment of functional recovery in a blind fashion, each animal was assigned a new code by an independent experimenter who was not involved in either animal surgery or functional assessment. All animals were pretrained for 1 week before surgery to become acquainted with open field and CatWalk gait analysis. Five rats were randomly selected from each group for behavioural testing 1 week to 12 months after surgery.

Basso, Beattie, and Bresnahan (BBB) locomotor score: Rats were allowed to walk freely in an open field, and the locomotor rating scale was determined after a 5‐min observation session (Basso et al., 1996).

CatWalk gait analyses: Gait analyses were performed using the CatWalk system as described in previous reports (Hamers, Koopmans, & Joosten, 2006). Rats were allowed to walk freely in an enclosed runway on a glass plate. Rat footprints were observed with a charge‐coupled device camera, followed by analyses with CatWalk XT 9.0 software (Noldus, Wageningen, Netherlands). Three uninterrupted runs were analysed for each time point. Three well‐established functional parameters were selected to analyse recovery after SCI: (a) pattern regularity, measured as a percentage of normal step sequence; (b) base of support, measured as the distance between the two hind paws perpendicular to the direction of walking; and (c) hind paw pressure, measured as the mean intensity of the contact area of the hind paw at the moment of maximal paw‐floor contact.

At 12 months post‐surgery, MEPs were recorded from five rats randomly selected from each group using previously published methods (Schlag, Hopf, & Redl, 2001). Briefly, following general anaesthesia, a stimulating electrode was placed on the skull surface of the cerebral cortex motor area or caudal to the SCI site (T12), a recording electrode was inserted into the gastrocnemius muscle of the contralateral hind limb, and a reference electrode was inserted subcutaneously between the two electrodes. The stimulating intensity was 10 mA (sham group) or 20 mA (other groups), and each site was stimulated twice successively, each with an interval of 1 min. MEPs were recorded using electromyography (MYTO EMG machine, Esaote, Italy), and the latency and amplitude of MEPs were evaluated.

### 2.5 Morphological investigation

Animals (three to five rats randomly selected from each group) were sacrificed 1 year after injury or implantation. Rats were deeply anaesthetized and transcardially perfused with PBS, followed by 4% paraformaldehyde (PFA) in 0.1 M of phosphate buffer (PB). Spinal cords were dissected, postfixed in PFA for 1 hr, and then placed in 30% sucrose in 0.1‐M PB at 4°C. For quantitative analysis, the spinal cord (length spanning 3‐mm rostral and caudal to epicentre of injury) was divided into six equal segments. Longitudinal sections were used for observation of axonal regeneration, whereas transverse sections for quantitative analyses were serially cut at 15 μm on a cryostat (Leica, Wetzlar, Germany). Every fifth section was mounted onto the same glass slide for immunolabelling. For immunohistochemistry, sections were washed in 0.01‐M PBS, permeabilized with 0.1% Triton X‐100, and blocked with bovine serum albumin and normal serum from the species of the appropriate secondary antibody. Next, sections were incubated for 12 hr with a primary antibody (Table 1) at 4°C, followed by incubation with a fluorescent‐labelled secondary antibody (Table 1) for 2 hr at room temperature. After counterstaining with Hoechst 33342 (1:1,000, Invitrogen, Carlsbad, CA), slides were cover slipped with Fluoromount G (Southern Biotech, Birmingham, AL). Sections were observed by confocal fluorescence microscopy (Leica, Wetzlar, Germany). Six sections from each segment were used for quantitative analyses. The number of axons in three random images (40× magnification) was counted for each section by observers who were blind to experimental groups and analysed using a Leica Qwin analytical system (Leica Imaging Systems, Ltd., Cambridge, England). Data are presented as the percentage of axons in each experimental group compared with the sham group (Sakai et al., 2012).

**Table 1:** Antibodies used for immunofluorescence and western blot

Antibody	Host	Dilution	Source
Anti‐5‐HT_1A_	Rabbit	1:1,000	Assay Designs, Ann Arbor, MI
Anti‐200‐kDa neurofilament (NF)	Mouse	1:800	Abcam, Cambridge, MA
Anti‐NeuN	Mouse	1:200	Abcam, Cambridge, MA
Anti‐CNPase	Mouse	1:400	Abcam, Cambridge, MA
Anti‐caspase‐3	Rabbit	1:1,000	Abcam, Cambridge, MA
Anti‐GFAP	Mouse	1:2,000	Abcam, Cambridge, MA
Anti‐caspase‐3 p17	Rabbit	1:500	Abcam, Cambridge, MA
Anti‐PARP	Rabbit	1:400	Abcam, Cambridge, MA
Anti‐cleave PARP	Rabbit	1:10,000	Abcam, Cambridge, MA
Anti‐β‐actin	Mouse	1:400	Beyotime, Shanghai
			
TRITC‐conjugated anti‐rabbit IgG	Goat	1:200	Sigma, Saint Louis, MO
TRITC‐conjugated anti‐mouse IgG	Goat	1:250	Sigma, Saint Louis, MO
FITC‐conjugated anti‐rabbit IgG	Goat	1:250	Sigma, Saint Louis, MO
FITC‐conjugated anti‐mouse IgG	Goat	1:250	Sigma, Saint Louis, MO
HRP‐labelled anti‐rabbit IgG	Goat	1:1,000	Beyotime, Shanghai
HRP‐labelled anti‐mouse IgG	Goat	1:1,000	Beyotime, Shanghai

For apoptosis analysis, specimens were collected from three rats per group (BMSC‐transplanted, PBS‐injected, z‐VAD‐fmk‐treated, or sham) at 3 weeks post‐surgery. Cell types undergoing apoptosis were determined by terminal deoxynucleotidyl transferase dUTP nick end labelling (TUNEL) and cell‐specific markers. Briefly, following TUNEL staining (remaining information was detailed in Supporting Information), sections were processed for immunocytochemistry using an antibody against neuron‐specific neuronal nuclei (NeuN), oligodendrocyte‐specific 2′,3′‐cyclic‐nucleotide 3′‐phosphodiesterase (CNPase), or astrocyte‐specific glial fibrillary acidic protein (GFAP)( Table 1). Likewise, double labelling with caspase‐3 and cell type‐specific markers was processed. For quantitative analysis, numbers of cells with TUNEL (or caspase‐3) and double‐labelling for each cell type‐specific marker were calculated from five random visual fields (40× magnification).

For transmission electron microscopy (TEM), three rats randomly selected from each group at 12 months post‐surgery were perfused with saline and a solution of 4% PFA/2.5% glutaraldehyde in 0.1‐M PB. After dissection, spinal cord samples containing the lesion site were fixed successively in 2.5% glutaraldehyde solution at 4°C for 2 hr and 1% osmic acid for 2 hr. Samples were then dehydrated in an ascending graded series of ethanol (50–100%), embedded overnight in Epon 812, and polymerized at 60°C for 48 hr. Embedded tissue was cut on an ultramicrotome (Leica, Heidelberg, Germany), and the resulting ultrathin slices were treated with lead citrate/uranyl acetate and examined by TEM (JEOL Ltd., Tokyo, Japan). Five randomized images for each section were analysed with a Leica Qwin analytical system. Numbers of myelinated nerve fibres and the thickness of myelin sheaths for regenerated nerve fibres were calculated.

### 2.6 Western blot analyses

Amounts of neurofilament protein (NF‐200) and serotonin 1A receptor (5‐HT_1A_) in each group 1 year after surgery, as well as caspase‐3 (p17 form), poly (ADP‐ribose) polymerase (PARP), and cleaved PARP in each group 3 weeks after surgery, were determined by electrophoresis and western blot analysis (remaining information was detailed in Supporting Information; Dwyer, Camacho, Kohanski, Callura, & Collins, 2012). Samples were collected from rats (*n* = 3) randomly selected from each group. Spinal cord tissue 3‐mm rostral and caudal to the epicentre was collected and lysed in radioimmunoprecipitation assay buffer (Beyotime, Haimen, China) for 30 min on ice. Tissue debris were pelleted by centrifugation at 12,000 rpm for 10 min at 4°C. Protein concentrations of tissue lysates were determined using a NanoPhotometer (Implen GmbH, Munich, Germany). Membranes (Millipore, Massachusetts, USA) were probed with 5‐HT_1A_ and NF‐200 antibodies, or caspase‐3 (p17), PARP, and cleaved PARP antibodies, followed by horseradish peroxidase‐conjugated secondary antibody (Table 1). β‐actin was used as a loading control. All western blotting experiments were repeated three times.

### 2.7 Statistical analyses

All data are expressed as mean ± standard deviation. Statistical evaluation was performed using Prism 6.0 (GraphPad Software, San Diego, CA). BBB scores and CatWalk footprint parameters were analysed with repeated measurement analysis of variance. Immunocytochemistry data for caspase‐3 were analysed with a Kruskal–Wallis test, and pairwise comparisons were performed using a Wilcoxon test with Bonferroni correction. Other experimental data were analysed with one‐way or two‐way analysis of variance, followed by least significant difference test for pairwise comparison. Values of *p* < 0.05 were considered statistically significant.

## 3 RESULTS

### 3.1 ST/MC + BMSCs implantation improved functional performance

**Figure 1:**
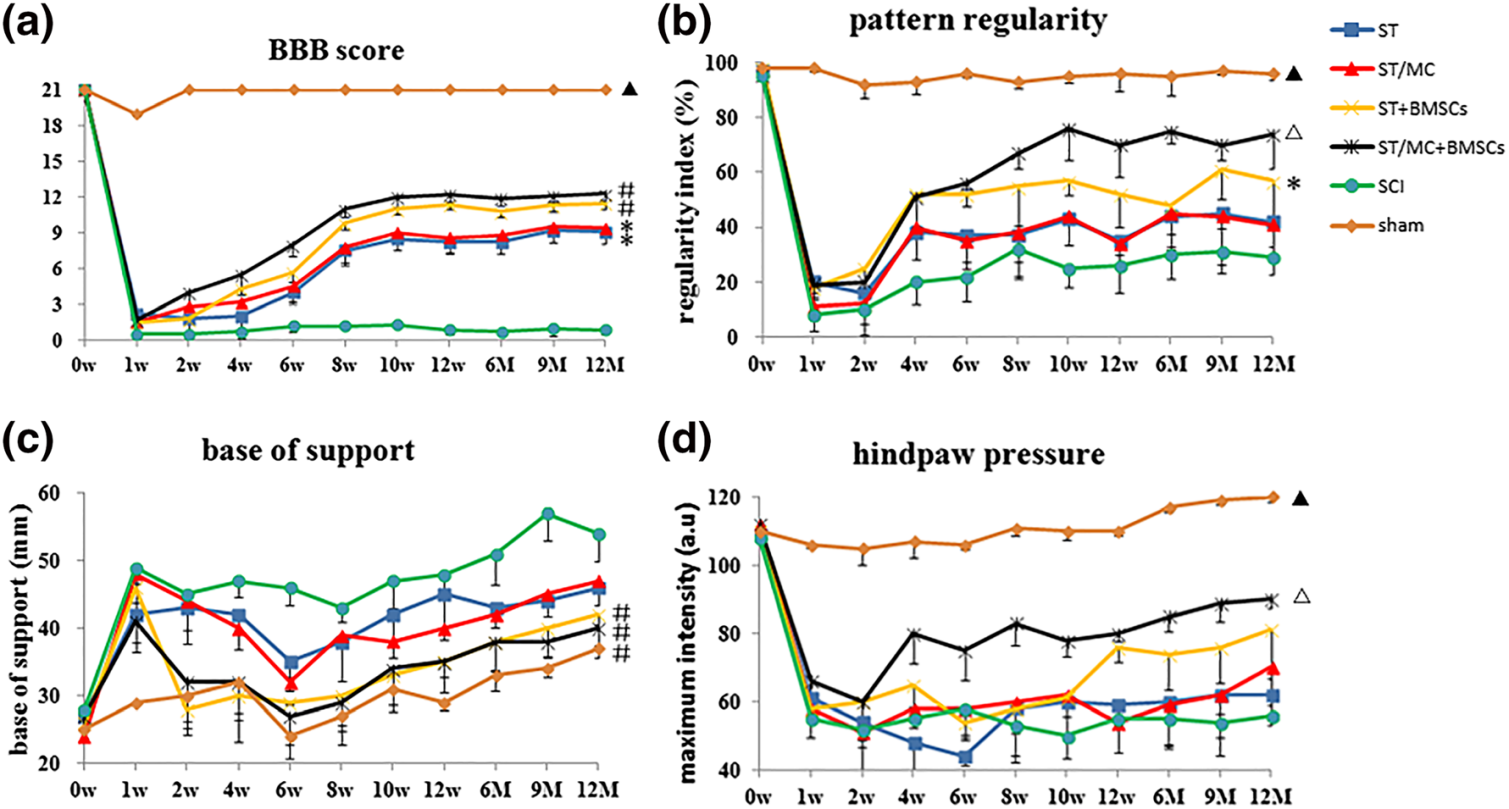
Locomotor function assessed with Basso, Beattie, and Bresnahan (BBB) score and CatWalk‐automated quantitative gait analysis. (a) BBB locomotor rating scale. (b–d) CatWalk assessment of three measures of walking as indicated, that is, pattern regularity, base of support, and hindpressure. The ST/MC + BMSCs group showed an overall better functional recovery compared with other groups. Data are expressed as mean ± standard deviation (*SD*; *n* = 5 rats per group) and analysed with repeated measures analysis of variance followed by LSD post hoc test. ^▲^ *p* < 0.05, versus any other group; ^△^ *p* < 0.05 versus SCI, ST, ST/MC, and ST + BMSCs; ^#^ *p*< 0.05 versus SCI, ST, and ST/MC; ^*^ *p* < 0.05 versus SCI. SCI, spinal cord injury (no repair); ST, O‐shaped single lumen tube; ST + BMSCs, ST seeded with BMSCs; ST/MC, H‐shaped single lumen tube with longitudinally aligned microchannels in the wall; ST/MC + BMSCs, ST/MC seeded with BMSCs in the H‐shaped lumen [Colour figure can be viewed at http://wileyonlinelibrary.com]

BBB locomotor score: At 1 week post‐surgery, the average BBB score was almost zero in all experimental groups, except the sham group. Notably, significant differences gradually emerged between the SCI group and ST, ST/MC, ST + BMSCs, and ST/MC + BMSCs groups. Animals in ST/MC + BMSCs and ST + BMSCs groups, having received scaffolds loaded with BMSCs, exhibited significantly improved BBB scores compared with SCI, ST, and ST/MC groups from 10 weeks to 12 months post‐surgery (*p* < 0.05; Figure 1a).

CatWalk gait analyses: Compared with other treatment groups and the SCI group, recovery of hind limb motor function in the ST/MC + BMSCs group was significantly enhanced. The regularity index was significantly reduced in all groups after injury, but that of the ST/MC + BMSCs group tended to increase compared with the other three implanted groups (*p* < 0.05) and the SCI group (*p* < 0.01) from 8 weeks post‐surgery (Figure 1b). Throughout the test period, the base of support of rats in ST/MC + BMSCs and ST + BMSCs groups was significantly lower compared with ST (*p* < 0.05), ST/MC (*p* < 0.05), and SCI (*p* < 0.01) groups; however, the sham group had the lowest scores (Figure 1c). Hind paw pressure was significantly increased in the ST/MC + BMSCs group compared with other groups from 4 weeks post‐surgery (*p* < 0.05). However, it did not begin to increase until Week 12 in the ST + BMSCs group and remained significantly lower than the ST/MC + BMSCs group until the end of the experiment (*p* < 0.05). Hind paw pressure was basically unchanged throughout the test period in ST, ST/MC, and SCI groups (Figure 1d).

**Figure 2:**
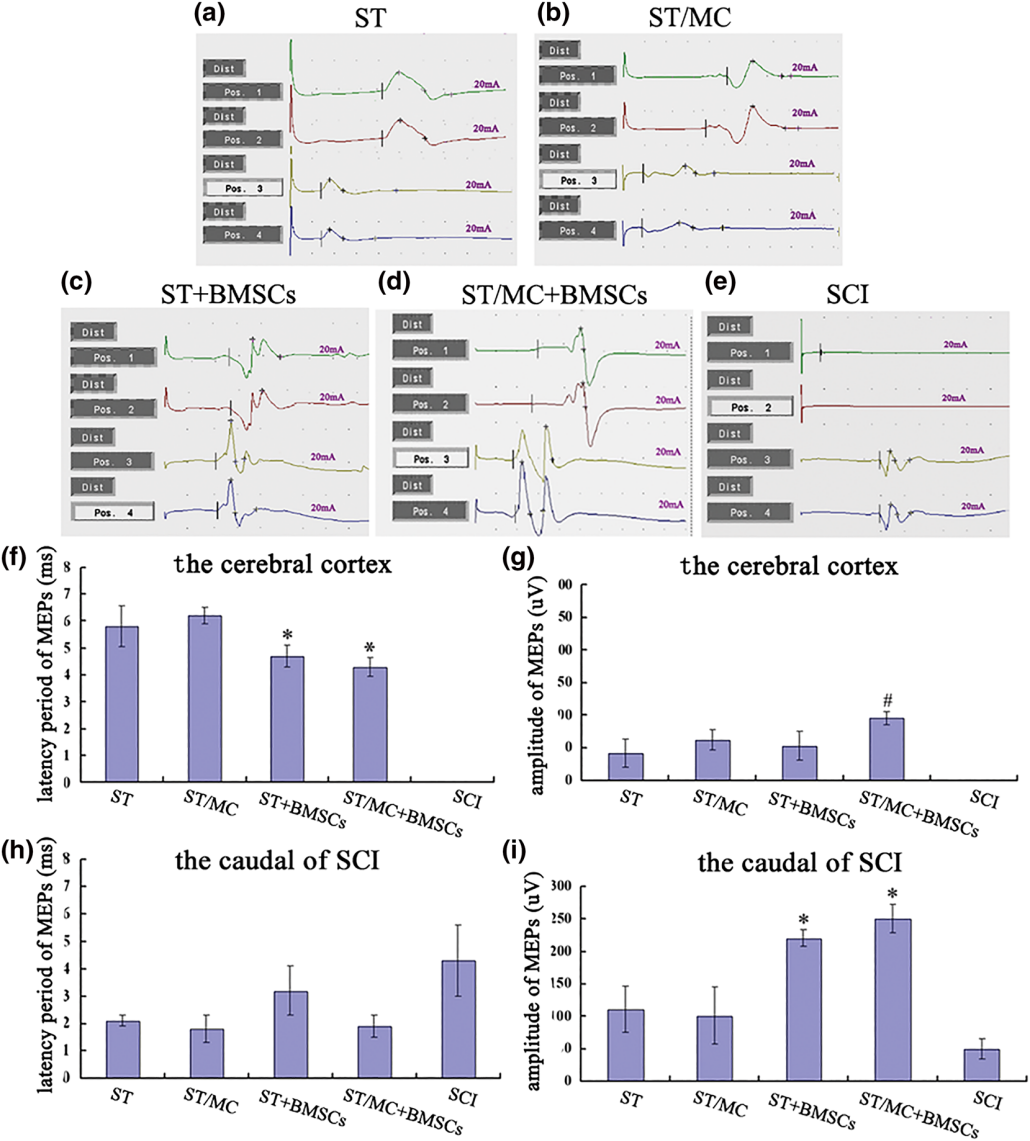
Motor evoked potentials (MEPs) recorded 12 months after SCI and repair. (a–e) Representative MEP traces recorded in the gastrocnemius muscle. Electrical stimulus was applied to either the motor cortex (Positions 1 and 2) or the spinal cord caudal to the lesion (Positions 3 and 4). (f–i) Bar charts showing latency and amplitude of MEPs when stimulated at indicated positions. Data are expressed as mean ± *SD* (*n* = 5 rats per group) and analysed with one‐way analysis of variance followed by LSD post hoc test. ^*^ *p* < 0.05 versus SCI, ST, and ST/MC; ^#^ *p* < 0.05 versus ST, ST/MC, and ST + BMSCs [Colour figure can be viewed at http://wileyonlinelibrary.com]

Twelve months after surgery, MEPs in the four bridged groups could be partially re‐recorded when stimulating the cerebral cortex or site caudal to the SCI site. However, in the SCI group, MEPs were only obtained with stimulation caudal of the SCI site (Figure 2a–e). In BMSC‐implanted groups, MEP latency was reduced when rats were stimulated in the cerebral cortex (*p* < 0.05; Figure 2f). In addition, MEP amplitudes in the ST/MC + BMSCs group were significantly increased compared with the other three bridged groups (*p* < 0.05; Figure 2g). When stimulating the area caudal area to the SCI site, there were no significant differences in MEP latency between any groups. However, MEP amplitudes in ST/MC + BMSCs and ST + BMSCs groups were significantly increased compared with ST/MC, ST, and SCI groups (*p* < 0.05; Figure 2h,i).

### 3.2 ST/MC + BMSCs allowed for descending motor fibre regrowth through the SCI lesion site

Twelve months after surgery, spinal cords were collected from rats in each group. In the four implanted groups, regenerated tissue filled the spinal cord defect, integrating the rostral and caudal ends of the spinal cord (Figure S2A–D). Conversely, for SCI group samples, no regenerated tissue was found between the two stumps, with the exception of residual dura mater.

**Figure 3:**
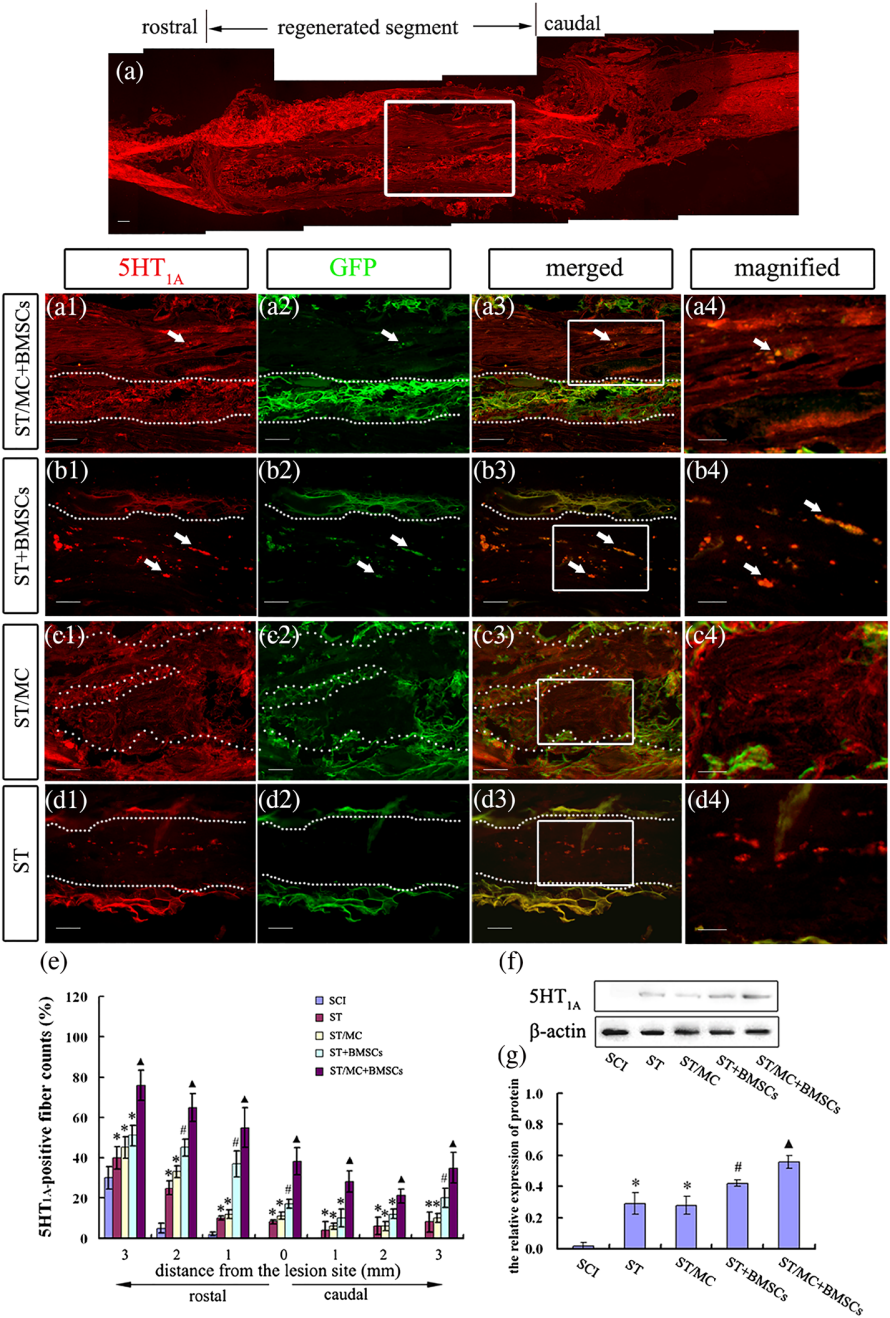
Regeneration of serotonergic axons into scaffolds at 12 months after SCI and repair. (a) An overview of longitudinal section of the repaired spinal cord immunostained for 5‐HT_1A_. (a1–a3) Magnified view of the boxed area in (a) showing colocalization of 5‐HT_1A_ with GFP‐expressing BMSCs. (b1‐b3) ST+BMSC_s_, (c1‐c3) ST/MC, and (d1‐d3) ST were found only a few randomly scattered 5‐HT1A+ dots. The chitosan/chitin tube wall (between the two dotted lines) exhibited stronger autofluorescence in the green channel than the red. The chitosan/chitin tube wall (between the two dotted lines) exhibited stronger autofluorescence in the green channel than the red. Specific GFP fluorescence was only observed in groups in which BMSCs were included, that is, ST/MC + BMSCs and ST + BMSCs, and partially colocalized with 5‐HT_1A_‐positive cells (arrows). Further magnified view of the boxed areas (b3‐d3) is shown (b4‐d4). (e) Bar chart showing counts of 5‐HT_1A_‐positive axons in the spinal cord 3 mm each rostral and caudal to the epicentre of injury. At least six sections per rat were evaluated. (f, g) Representative western blots of spinal cord tissue at 3 mm each rostral and caudal to the epicentre of injury and bar chart showing densitometry of the blots. Data are expressed as mean ± *SD* (*n* = 5 rats per group for (e) and 3 for (g)) and analysed with two‐way (e) and one‐way (g) analysis of variance followed by LSD post hoc test. ^▲^ *p* < 0.05 versus SCI, ST, ST/MC, and ST + BMSCs; ^#^ *p* < 0.05 versus SCI, ST, and ST/MC; ^*^ *p* < 0.05 versus SCI. Scale bar: 100 μm (a); 50 μm (a1–a3, b1–b3, c1–c3, d1–d3); 20 μm (a4, b4, c4, d4) [Colour figure can be viewed at http://wileyonlinelibrary.com]

The raphespinal axon is the main nerve fibre mediating locomotor function in the spinal cord (Ghosh & Pearse, 2014). Serotonin (5‐HT) is a neurotransmitter specifically transmitted by raphespinal axons in the spinal cord. Immunolabelling indicated a few 5‐HT_1A_‐positive (5‐HT_1A_^+^) nerve fibres regenerated into and extended throughout the implants of ST/MC + BMSCs group rats, yielding an almost linear arrangement of axons (Figure 3a, a1–a4). However, in ST + BMSCs, ST/MC, and ST groups, only a few randomly scattered 5‐HT_1A_^+^ dots were found (Figure 3b–d). Notably, a few transplanted GFP‐BMSCs in ST + BMSCs and ST/MC + BMSCs groups were also found to be positive for 5‐HT_1A_ (Figure 3a3,a4,b3,b4). Quantification of 5‐HT_1A_^+^ nerve fibres from 3‐mm rostral to 3‐mm caudal areas in the transverse lesion indicated a significantly increased number of 5‐HT_1A_^+^ axons in the ST/MC + BMSCs group compared with the other three implanted groups (*p* < 0.05; Figure 3e). In transverse sections, percentages of 5‐HT_1A_^+^ nerve fibres at the epicentre of ST/MC + BMSCs, ST + BMSCs, ST/MC, and ST groups were 38.2% ± 6.7%, 17.5% ± 2.3%, 11.5% ± 1.8%, and 8.7% ± 0.8%, respectively (*p* < 0.05). In western blot analysis, significantly increased levels of 5‐HT_1A_ were observed in the ST/MC + BMSCs group compared with other implantation groups (*p* < 0.05; Figure 3f,g).

**Figure 4:**
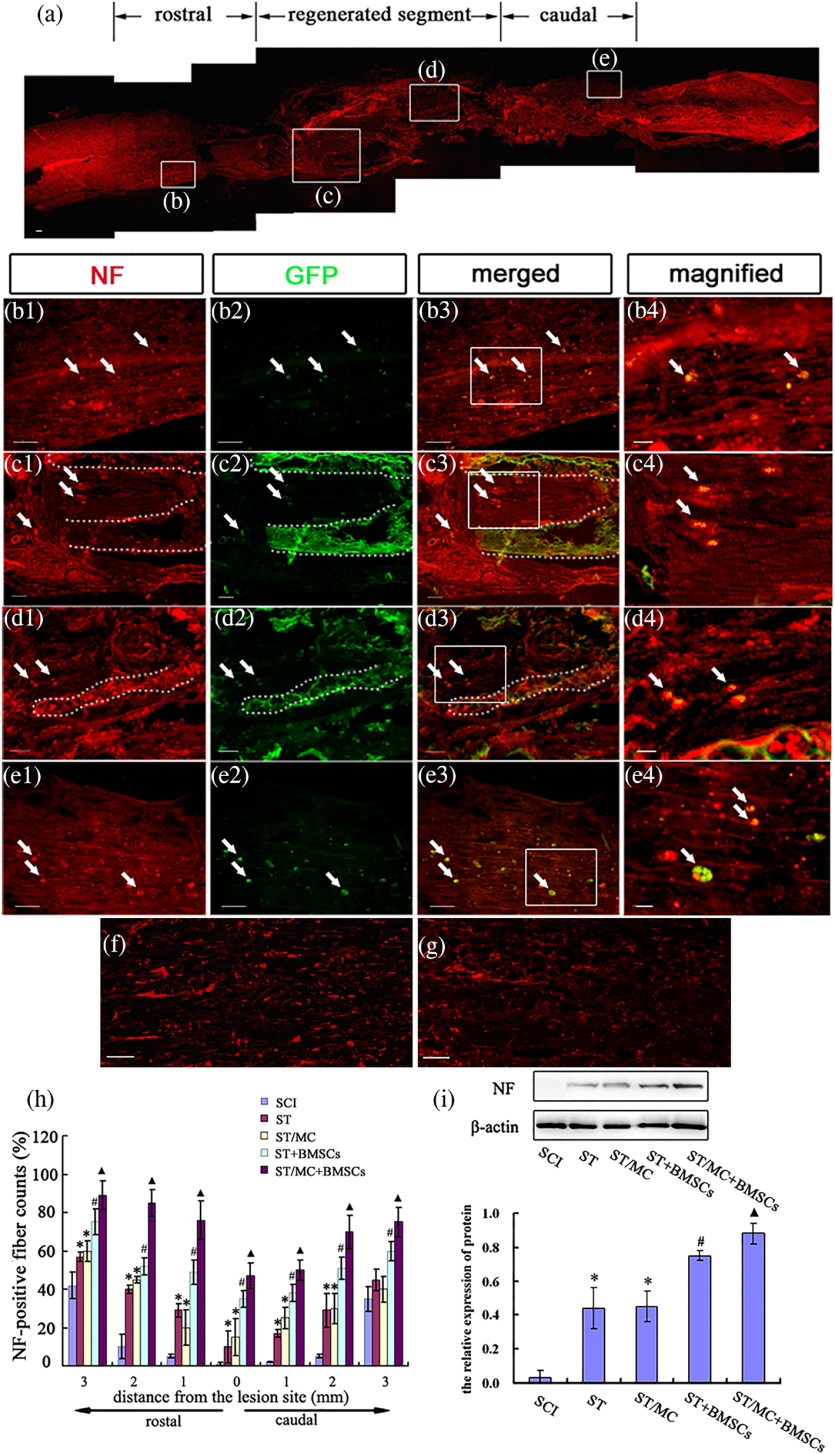
Axonal regeneration into ST/MC scaffolds seeded with BMSCs at 12 months after SCI and repair. (a) An overview of longitudinal section of the repaired spinal cord immunostained for NF‐200, an axonal marker. (b1–e4) Magnified view of the boxed area in (a). Arrows show colocalization of NF‐200 with GFP‐expressing BMSCs. Dotted lines outline edges of the debris of the degraded microchannels or lumen. Further magnified view of the boxed areas (b3‐e3) is shown (b4‐e4). (f, g) NF‐200‐positive nerve fibres well aligned along the longitudinal axis in microchannels (f) but less organized in the tube lumen (g). (h) Bar chart showing axonal counts in the spinal 3 mm each rostral and caudal to the epicentre of injury. At least six sections per rat were evaluated. (i) Representative western blots of spinal cord tissue at 3 mm each rostral and caudal to the epicentre of injury and bar chart showing densitometry of the blots. Data are expressed as mean ± *SD* (*n* = 5 rats per group for (h) and 3 for (i)) and analysed with two‐way (h) and one‐way (i) analysis of variance followed by LSD post hoc test. ^▲^ *p* < 0.05 versus SCI, ST, ST/MC, and ST + BMSCs; ^#^ *p* < 0.05 versus SCI, ST, and ST/MC; ^*^ *p* < 0.05 versus SCI. Scale bar: 100 μm (a); 50 μm (b1–b3, c1–c3, d1–d3, e1–e3); 20 μm (b4, c4, d4, e4); 25 μm (f, g) [Colour figure can be viewed at http://wileyonlinelibrary.com]

NF‐200^+^ nerve fibres in the entire defective segment were observed at 12 months post‐surgery in the ST/MC + BMSCs group (Figure 4a). Some transplanted GFP‐BMSCs colocalized with NF‐200 were detected among the nerve fibres at the rostral end of the injured spinal cord (Figure 4b1–b4). In the middle segment of regenerated tissue, some nerve fibres were arranged linearly along the debris of the degraded microchannel (Figure 4c1–c4,d1–d4,f), whereas others were random or formed connected networks within the H‐shaped lumen (Figure 4c1–c4,d1–d4,g). A few GFP‐BMSCs that were NF‐200^+^ differentiated into neurons that migrated to the caudal parenchyma (Figure 4e1–e4). Furthermore, quantitative determination of NF‐200^+^ nerve fibres from 3‐mm rostral to 3‐mm caudal areas of the transected lesion sites in each group was conducted. In seven transverse sections, NF‐200^+^ fibres were significantly increased in the ST/MC + BMSCs group compared with the other groups (*p* < 0.05; Figure 4h). In addition, the results of western blotting showed that NF‐200 expression in all groups was consistent with the results of immunohistochemistry; that is, it appeared higher in the ST/MC + BMSCs group than in any other group (Figure 4i).

TEM revealed that there were more axon fascicles for myelinated or unmyelinated nerve fibres in the midportion of regenerated tissues in ST/MC + BMSCs and ST + BMSCs groups (Figure S3A,B,D,E). However, there was a thicker, more uniform and electron‐dense lamellar myelin sheath on myelinated nerve fibres in the ST/MC + BMSCs group compared with the ST + BMSCs group. Moreover, synapse‐like connections were detected (Figure S3C), and new capillaries with well‐established ultrastructure were also readily observed (Figure S3F). Only a small number of newly formed axons with or without myelin sheaths were surrounded by fibroblasts and collagen fibres in ST and ST/MC groups (Figure S3G,H), whereas many fibroblasts and collagen fibres formed scar tissue without regenerated axons on stumps in the SCI group (Figure S3I). The number of myelinated nerve fibres in the ST/MC + BMSCs group (11.0 ± 1.3) was significantly higher than all other groups (*p* < 0.05; Figure S3J). However, there were no significant differences in myelin sheath thickness among the four implanted groups (Figure S3K).

### 3.3 BMSCs alleviated neural apoptosis after SCI

With the exception of the sham group, TUNEL demonstrated extensive apoptosis in each group at 1 week post‐surgery, with no significant difference in numbers of TUNEL^+^ cells between the three experimental groups (Figures S4 and S5). This suggested that massive apoptosis occurred immediately after SCI. TUNEL^+^ cells in ST/MC + BMSCs and ST/MC + z‐VAD‐fmk groups were relatively reduced at 2 weeks post‐surgery, whereas a large number of apoptotic cells still existed in the ST/MC + PBS group (Figures S4 and S5). Numbers of TUNEL^+^ cells in ST/MC + BMSCs and ST/MC + z‐VAD‐fmk groups were significantly reduced compared with the ST/MC + PBS group at 2 and 3 weeks post‐surgery (*p* < 0.05; Figures S4 and S5). Apoptotic cells in ST/MC + BMSCs and ST/MC + z‐VAD‐fmk groups were significantly decreased at 3 weeks compared with 1 week after surgery (*p* < 0.05). However, numbers of apoptotic cells in the ST/MC + PBS group were not significantly decreased within 3 weeks (Figures S4 and S5).

**Figure 5:**
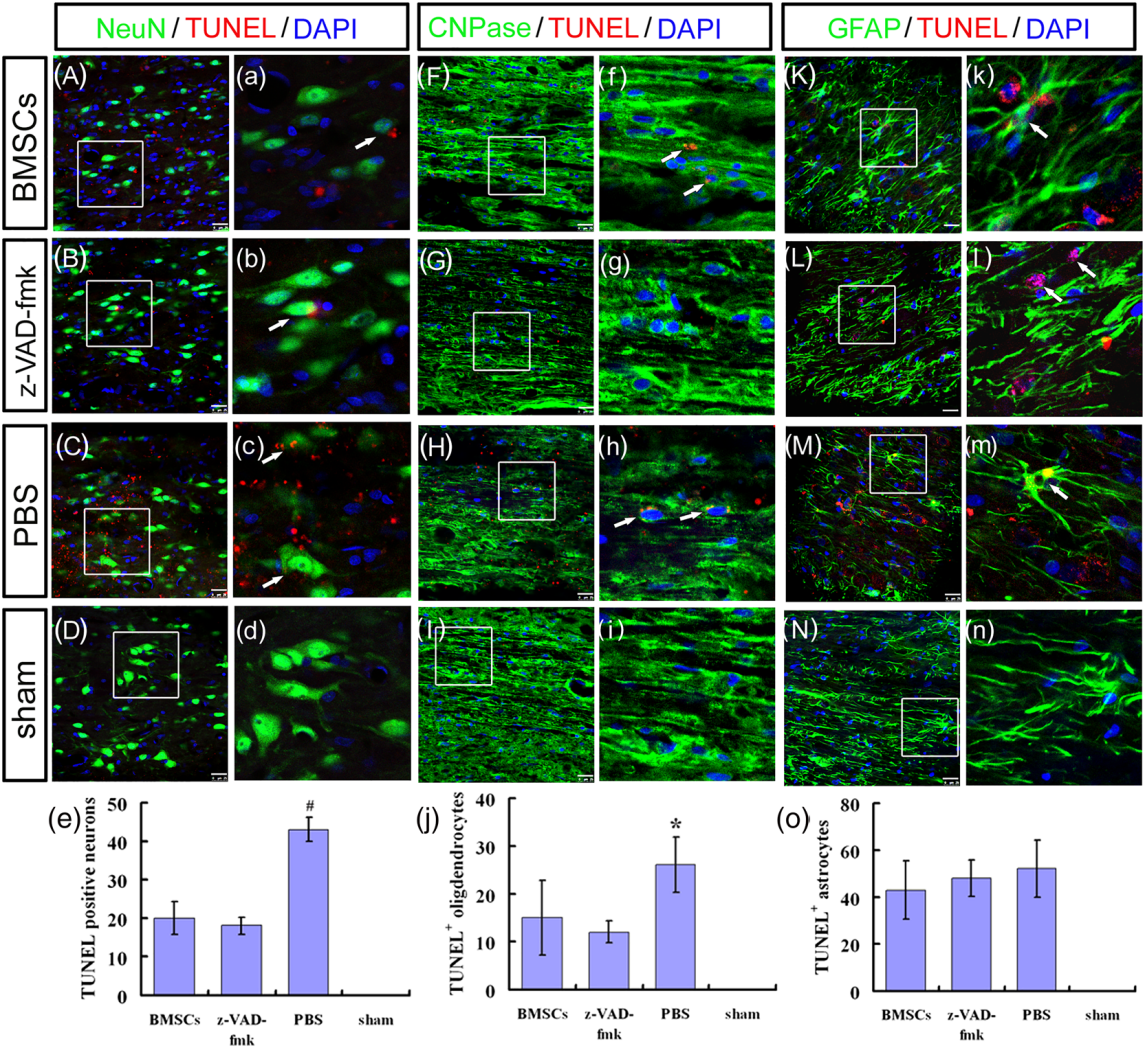
BMSCs seeded in tube with H‐shaped lumen and microchannels inhibit apoptosis of neurons and oligodendrocytes 3 weeks after repair of SCI. Shown are representative images and quantification of immunofluorescence staining to assess apoptosis of neurons (A–e), oligodendrocytes (F–j), and astrocytes (K–o). As a pan‐caspase inhibitor, z‐VAD‐fmk was injected into ST/MC to serve as a control group. Images labelled with lower‐case alphabets showed magnified view of the boxed area in corresponding images labelled with the upper case. TUNEL, NeuN, CNPase and GFAP are markers for apoptotic cells, neurons, oligodendrocytes, and astrocytes, respectively. DAPI is a nuclear marker. At least six sections for each rat were immunostained and evaluated. Data are expressed as mean ± *SD* (*n* = 3 rats per group) and analysed with one‐way analysis of variance followed by LSD post hoc test. ^*^ *p* < 0.05 versus ST/MC + z‐VAD‐fmk; ^#^ *p*< 0.01 versus ST/MC + BMSCs, ST/MC + z‐VAD‐fmk. Scale bar: 50 μm (A–D, F–I, K–N); 20 μm (a–d, f–i, k–n) [Colour figure can be viewed at http://wileyonlinelibrary.com]

Three weeks after injury, many cells expressing NeuN, CNPase, or GFAP colocalized with TUNEL were detected in the ST/MC + PBS group (Figure 5C,H,M,c,h,m), thus demonstrating the diversity of apoptosis after SCI. However, engrafted ST/MC + BMSCs or ST/MC + z‐VAD‐fmk significantly decreased TUNEL staining of neurons and oligodendrocytes (Figure 5A,B,F,G,a,b,f,g). Indeed, numbers of NeuN^+^/TUNEL^+^ apoptotic neurons were significantly lower in ST/MC + BMSCs and ST/MC + z‐VAD‐fmk groups compared with the ST/MC + PBS group (*p* < 0.01; Figure 5e). This result indicated that use of BMSCs in tissue engineering could reverse SCI‐induced neuronal apoptosis, as in z‐VAD‐fmk. Similarly, numbers of CNPase^+^/TUNEL^+^ cells in the ST/MC + z‐VAD‐fmk group were also lower than observed in the ST/MC + PBS group (*p* < 0.05), but there was no significant difference between ST/MC + BMSCs and ST/MC + PBS groups or ST/MC + BMSCs and ST/MC + z‐VAD‐fmk groups (Figure 5j). GFAP^+^/TUNEL^+^ cells were observed in the other three groups, but not the sham group; however, there was no significant difference in the number of double‐labelled cells between these three groups (Figure 5K–o,k–n).

**Figure 6:**
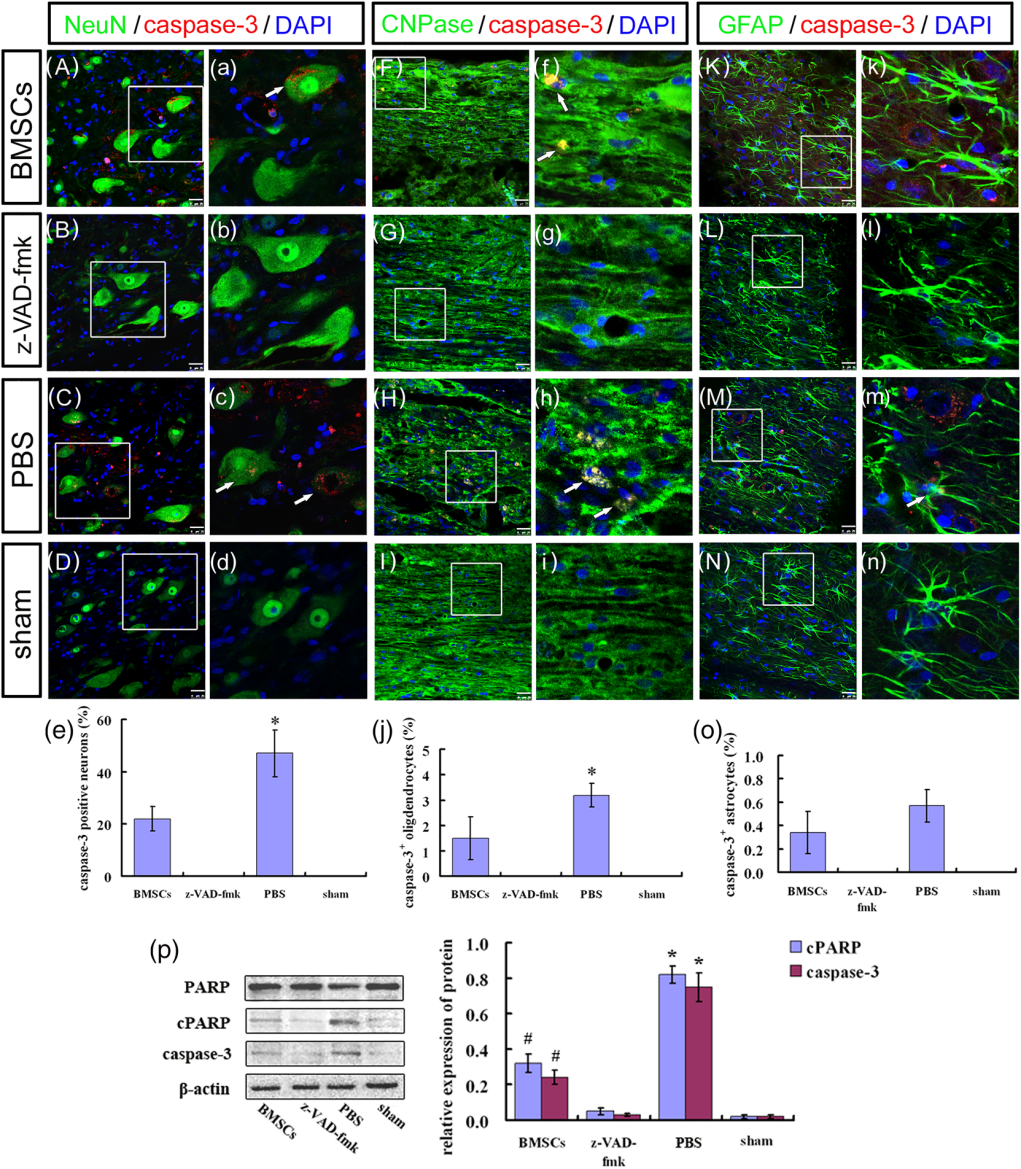
BMSCs seeded in tube with H‐shaped lumen and microchannels reduce the expression of caspase‐3 in neurons and oligodendrocytes 3 weeks after repair of SCI. (A–o) Representative images and quantification of immunofluorescence staining to assess expression of caspase‐3 in neurons (A–e), oligodendrocytes (F–j), and astrocytes (K–o). Images labelled with lower‐case alphabets showed magnified view of the boxed area in corresponding images labelled with the upper case. At least six sections for each rat were immunostained and evaluated. (p) Representative western blots and densitometry showing the protein expression level of cleaved poly (ADP‐ribose) polymerase (cPARP) and caspase‐3. Total PARP was also shown. β‐actin was used as a loading control. Data are expressed as mean ± *SD* (*n* = 3 rats per group) and analysed with Kruskal–Wallis test followed by Wilcoxon test with Bonferroni correction (e, j, o) or two‐way analysis of variance followed by LSD post hoc test (p). ^*^ *p* < 0.05 versus ST/MC + BMSCs (e, j ); ^*^ *p* < 0.05 versus ST/MC + BMSCs, ST/MC + z‐VAD‐fmk, and sham (p); ^#^ *p* < 0.05 versus ST/MC + z‐VAD‐fmk and sham (p). Scale bar: 50 μm (A–D, F–I, K–N); 25 μm (a–d); 20 μm (f–i, k–n) [Colour figure can be viewed at http://wileyonlinelibrary.com]

In addition, numbers of caspase‐3^+^/NeuN^+^ cells and caspase‐3^+^/CNPase^+^ cells in the ST/MC + BMSCs group were lower than observed in the ST/MC + PBS group (*p* < 0.01 and *p* < 0.05, respectively; Figure 6A,a,C,c,e,F,f,H,h,j). Moreover, there was no significant difference in the number of caspase‐3^+^/GFAP^+^ cells between ST/MC + BMSCs and ST/MC + PBS groups (Figure 6K,k,M,m,o). Notably, there was no caspase‐3 expression at all in ST/MC + z‐VAD‐fmk and sham groups (Figure 6B,D,G,I,L,N). With regard to expression of caspase pathway members caspase‐3 p17, PARP, and cleaved PARP, expression of caspase‐3 p17 and cleaved PARP was lower in the ST/MC + BMSCs group compared with the ST/MC + PBS group (*p* < 0.05) but was higher compared with ST/MC + z‐VAD‐fmk and sham groups (*p* < 0.05), in which PARP expression of each group was basically unchanged (Figure 6p).

## 4 DISCUSSION

The results of this study showed that a novel integrated scaffold combining a single H‐shaped central lumen with many surrounding microchannels on the tubular wall (to mimic the spinal cord architecture) provided partitioned regenerative regions for white and grey matter after SCI. Moreover, when the scaffold was loaded with BMSCs, the number of regenerating axons was further increased. Tissue engineering with ST/MC and BMSCs had the potential to promote spinal cord regeneration through mechanical guidance by scaffolds, antiapoptotic effects of BMSCs, and differentiation of a few BMSCs into neurons.

The macrostructure and microstructure of biomaterial scaffolds, as well as their chemical properties, are important factors influencing the quality of tissue engineering and subsequent repair. In previous decades, different scaffold macroarchitectural designs were found to facilitate axonal growth across a lesion after SCI (Madigan et al., 2009; Raspa, Pugliese, Maleki, & Gelain, 2016; Tsintou, Dalamagkas, & Seifalian, 2015). For instance, biomaterial polymers, such as gels, may be suitable for filling for contusions or minor tears of the spinal cord (Donoghue et al., 2013). However, an ST, multiple channels, and numerous microchannels better serve to fill in larger defects by bridging gaps and traversing the glial scar or cystic cavities (Stokols et al., 2006; Moore et al., 2006; Novikova et al., 2018; Liu et al., 2017). An ST with a large “O” shape lumen may facilitate the migration of cells or tissue regeneration, which is especially suitable for grey matter regeneration containing neuronal somata and a complex horizontal neural network (Nomura, Katayama, Shoichet, & Tator, 2006). We also found it necessary to increase the lumen diameter when the rostral and caudal spinal cord stumps were inserted into the lumen, as this caused the spinal canal space to fail to accommodate. At the same time, when the two spinal cord stumps were lifted up and inserted into the lumen of the thicker tubular wall, it caused further injury. Importantly, if the injured spinal cord stumps were attached end to end to the tube, it might prevent white matter axons from elongating, as the thick tubular wall lies just in the path of elongation for white matter tracts. Wong et al. (2008) found that an open path with or without a core can improve regeneration of the severed spinal cord compared with a cylinder, tube, or multiple channels. From another viewpoint, it has also been shown that tubular wall thickness, especially dense walls, will hinder the axon ingrowth of white matter. Given the linear arrangement of nerve fibre tracts in white matter, the addition of many microchannels along the longitudinal axis of the tubular wall of the scaffold might solve this problem. Indeed, a few studies investigating the use of numerous microchannels demonstrated good results for repair of dorsal or lateral hemisection SCI, especially axonal tracts in white matter (Günther et al., 2015; Liu et al., 2017; Madaghiele, Sannino, Yannas, & Spector, 2008; De Laporte et al., 2009; Stokols & Tuszynski, 2006; Chedly et al., 2017). One major advantage of scaffolds with numerous microchannels is that they are well suited for the regeneration of both descending and ascending axonal tracts in white matter (Gros, Sakamoto, Blesch,Havton, & Tuszynski, 2010; Günther et al., 2015).

In this study, a new integrated scaffold consisting of a single central tube and many surrounding microchannels allowed the migration and growth of cells and axons in the large H‐shaped central lumen of the ST/MC scaffold. Although this usually occurred in the form of random or connected networks, some axons also extended into the microchannels of the ST/MC scaffold wall. Most notably, a few 5‐HT_1A_^+^ nerve fibres were observed to be linearly arranged in residual microchannel debris. Although the ST scaffold could bridge spinal cord defects and facilitate axon extension to a certain extent, the path of regenerated axons in the large lumen of the ST was usually random and disordered, and the dense scaffold wall prevented axons in white matter from elongating. However, our results showed that the large lumen of the ST/MC scaffold allowed for nerve cell migration as well as transverse connections between cells, whereas the many oriented microchannels on the ST/MC scaffold wall appeared to be more suitable for linear extension of the descending and ascending axonal tracts of white matter. Importantly, the diameter of the microchannels impacts axonal regeneration. As previous reports on microchannels for SCI repair employed microchannel diameters in the range of a few dozen microns to 450 μm (Günther et al., 2015; Xiong et al., 2009; De Laporte et al., 2009; Stokols & Tuszynski, 2004; Krych et al., 2009; Pawar et al., 2015), the diameter of microchannels used in this study was 250 μm.

In this study, the second strategy to enhance axonal regeneration was seeding of the ST/MC scaffold with BMSCs, based on cell therapy (Assinck et al., 2017; Kim et al., 2018). When BMSC‐loaded ST/MC scaffolds were used to bridge spinal cord defects, we found that they had a significant effect on neuroregeneration. In spinal cord samples, including the entire defect gap, continuous NF‐200^+^ nerve fibres were found in the ST/MC + BMSCs group. Some fibres were arranged in a linear pattern parallel to the debris in the degraded microchannels. More significantly, the few linear axonal tracts in regenerated tissue extending throughout the microchannels were 5‐HT_1A_^+^, implying that some raphespinal axons were repaired, which may improve locomotor recovery (Hong et al., 2017). In addition, a small number of 5‐HT_1A_^+^/GFP^+^ or NF‐200^+^/GFP^+^ cells and fibres were found in the ST/MC + BMSCs group, especially regenerating 5‐HT_1A_^+^ axons, indicating that some BMSCs could differentiate into neurons. The results were similar to those previously reported for the treatment of SCI with BMSC transplantation, which found that human mesenchymal precursor cell transplantation could promote serotonergic or raphespinal axon growth and functional recovery (Hodgetts,Simmons, & Plant 2013; Mannoji et al., 2014; Onuma‐Ukegawa et al., 2015). Significantly increased numbers of 5‐HT_1A_^+^ or NF‐200^+^ axons were observed from 3‐mm rostral to 3‐mm caudal areas within the transected lesion site in the ST/MC + BMSCs group compared with the other three implanted groups. Western blot analysis of 5‐HT_1A_ and NF‐200 levels showed the same results. In addition, TEM images and quantitative analyses supported the above results.

With regard to effects of the ST/MC scaffold and BMSCs for regeneration of spinal cord tissue, reconnection of a few descending motor tracts led to partial recovery of locomotion function in rats. This was matched by a significant improvement of MEPs for descending motor pathways, as well as locomotion scores in the BBB test or CatWalk gait analyses. Notably, in the CatWalk gait analyses, we also noticed that data for the base of support abnormally increased at 6 weeks post‐surgery, including in the sham group. We speculated that this might be related to significant weight gain by rats.

Theoretically, inclusion of a BMSCs alone group would help emphasize the need for scaffolds in the repair of a spinal cord defect. However, practically, such a group is not included in the present study or previous studies in the literature, owing to lack of matrix carrier for the cells to reside and contact with the injured spinal cord tissue (Günther et al., 2015; Ritfeld et al., 2014).

The issue of level off of BBB score after 3–4 months after SCI is intriguing. To the best of our knowledge and postulation, it could be attributable to the effect of implanted cells and the intrinsic growth capacity of the spinal cord tissue. On one hand, the capability of implanted cells in promoting regeneration may not last long, which has also been indicated in a previous study (Liu et al., 2019). On the other hand, it is possible that functionally potent regeneration might occur only in the early stage after SCI (Hodgetts et al., 2013). Unravelling of the underlying mechanisms in future studies may lead to a better formula of cell implantation and/or other treatment that can yield improved therapeutic effect.

As a seed cell, only a few BMSCs differentiated into neurons and glia in the SCI micro‐environment to replenish nerve tissue. As such, we questioned whether BMSCs in the ST/MC scaffold could act similarly to direct BMSC transplantation to protect nerve tissue, such as by reducing apoptosis caused by secondary injury after SCI. We found that the implantation of BMSCs on ST/MC scaffolds could suppress SCI‐induced apoptosis of neurons and oligodendrocytes, thus eliciting a certain protective effect on axons and myelin sheaths in the region surrounding the epicentre. However, we did not find that BMSCs significantly inhibited the apoptosis of astrocytes in our experiment. This is in contrast to a report by Sakai et al. (2012), who found that transplantation of stem cells from human exfoliated deciduous teeth (similar to BMSCs) after SCI could strongly suppress the apoptosis of astrocytes. Further examination of this difference is needed, but the same result occurred when we employed the pan‐caspase inhibitor z‐VAD‐fmk as a positive control. We also found that BMSCs could reduce the apoptosis of neurons and oligodendrocytes after SCI by blocking the expression of caspase‐3, an important molecule involved in the apoptosis pathway after nerve tissue injury. These results suggested that BMSCs loaded in the scaffold could reduce secondary injury through strong neuroprotection of neurons and oligodendrocytes, and those effects might be more significant than seed cell differentiation.

So how did BMSCs protect neural tissue against further damage? Our previous report indicated that spinal cord tissue and BMSCs show a two‐way effect in coculture, in which BDNF secretion is significantly promoted. Thus, BMSCs could secrete neurotrophic factors to protect neurons and promote neurite outgrowth, whereas spinal cord tissue could secrete soluble factors to induce differentiation of BMSCs into neurons (Lin et al., 2014). These results have also been partially confirmed by in vivo experiments (Han et al., 2015; Ritfeld et al., 2015). In addition, BDNF can reportedly prevent apoptosis of neurons and oligodendrocytes after SCI, thereby protecting the spinal cord from secondary injury (Nakajima et al., 2010). These clues likely explain some of the mechanisms employed by BMSCs in scaffolds to promote regeneration of damaged neural tissue.

Of course, because of the complexities of SCI, successful repair is difficult to achieve with a single‐component treatment. Therefore, we intend to achieve precision regeneration by applying diverse kinds of seed cells and/or cytokines to different regions of ST/MC scaffolds to form distinct micro‐environments that meet the regeneration needs of different spinal cord regions. To do this, the optimal position of microchannels must also be determined, such that it corresponds to the position of each white matter tract.

## 5 CONCLUSIONS

A novel integrated scaffold with a single H‐shaped central lumen and many surrounding microchannels was loaded with BMSCs in H‐shaped central lumen before transplantation to bridge long complete transection defects of rat thoracic spinal cords. ST/MC scaffolds provided partitioned grey and white matter regeneration; in particular, a few 5‐HT_1A_^+^ nerve fibres were linearly arranged. When a BMSC‐loaded scaffold was employed, the number of regenerating axons was further increased. In addition, BMSCs elicited an antiapoptosis effects and differentiated into neurons. Future studies will apply diverse kinds of seed cells and/or cytokines to different regions of the scaffold. Indeed, by exploiting the characteristics of the ST/MC and forming distinct micro‐environments suitable for regeneration of different parts of the spinal cord, we can achieve precision regeneration.

## CONFLICT OF INTERESTS

There are no conflicts to declare.

## CONFLICT OF INTEREST

The authors have declared that there is no conflict of interest.

## Supporting information


**Figure S1.** **ST and ST/MC for repairing SCI.** (A) Schematic diagram of ST design and size, a central empty area and surrounding solid ring represented the circular lumen and wall of the tube, respectively. (B) Schematic diagram of ST/MC design and size, an ‘H’ shape central lumen and surrounding solid tubular wall correspond to the spinal cord grey matter and white matter, respectively; moreover, many microchannels on the tubular wall. (C) ST and (D) ST/MC micrograph on SEM, Scale bar: 2 mm
**Figure S2. Scaffold with or without BMSCs bridged SCI defect for 12 months.** Low power overview of the spinal cord of an animal grafted with ST (A), ST/MC (B), ST + BMSCs (C), or ST/MC + BMSCs (D) bridged to a 5 mm defect at complete transverse thoracic spinal cord for 1 year, regenerated tissue filled the spinal cord defect, integrating the rostral and caudal ends of the spinal cord
**Figure S3. TEM images of nerve fibers in all groups at 12 months SCI.** (A) There were more axon fascicles for myelinated or unmyelinated nerve fibers in the mid‐portion of the regenerated tissues in the ST/MC + BMSCs group. (B) Higher magnifications of area boxed in (A), axons of myelinated nerve fibers wrapped by a thick, uniform and electron‐dense lamellar myelin sheath. (C) There were synapse‐like connections in the regenerated tissues of ST/MC + BMSCs. (D) There were some myelinated or unmyelinated nerve fibers of the regenerated tissues in the ST + BMSCs group, (E) higher magnifications of area boxed in (D). (F) The new capillaries with a well‐established ultrastructure of the regenerated tissues were in the ST + BMSCs and ST/MC + BMSCs groups. (G) and (H) Only a small number of newly formed axons with or without myelin sheath were surrounded by fibroblasts and collagen fibers in the ST and ST/MC groups. (I) There was only glial scar in the SCI group. (J) Bar chart showed significant higher in the number of the myelinated nerve fibers of the ST/MC + BMSCs group. (K) Bar chart showed no significant differences in the thickness of the myelin sheath among 4 implanted groups. (J and K: mean ± SD; ^▲^ *p* < 0.05 vs. ST, ST/MC, and ST + BMSCs; * *p* < 0.05 vs. ST and ST/MC; by one‐way ANOVA analysis of variance, followed by an LSD‐t test pairwise comparison; *n* = 3 rats per group, > 3 sections per rat). Scale bar: 5 μm (H, I); 2 μm (A, D, F, G); 1 μm (B, E); 0.5 μm (C)
**Figure S4. BMSCs alleviating neural apoptosis during 1‐3 week after SCI.** The number of TUNEL^+^ cells in the ST/MC + BMSCs and ST/MC + z‐VAD‐fmk groups was significantly lower than that in the ST/MC + PBS group at 2 and 3 weeks after surgery, respectively. The apoptosic cells in the ST/MC + BMSCs and ST/MC + z‐VAD‐fmk groups were significantly decreased at 3 weeks than that at 1 week after surgery, however, that of the ST/MC + PBS group were not significantly decreased within 3 weeks. (mean ± SD; * *p* < 0.05 vs. ST/MC + PBS; ^#^ *p* < 0.05 vs. ST/MC + BMSCs and ST/MC + z‐VAD‐fmk 1w after surgery; by two‐way ANOVA analysis of variance, followed by an LSD‐t test pairwise comparison; *n* = 3 rats per group, > 6 sections per rat)
**Figure S5. BMSCs alleviating neural apoptosis during 1 to 3 week after SCI.** TUNEL labeling (red) showed extensive apoptosis in each group at 1 week after SCI, and there was no significant difference in TUNEL^+^ cell number between 3 groups (A‐C). TUNEL^+^ cells in the ST/MC + BMSCs and ST/MC + z‐VAD‐fmk groups were relatively reduced during 2‐3 week after SCI, while a large number of apoptotic cells still existed in the ST/MC + PBS group (D‐F, G‐I). Scale bar: 50 μm
