# Supplementary figures and images for: Tubular scaffold with microchannels and an H‐shaped lumen loaded with bone marrow stromal cells promotes neuroregeneration and inhibits apoptosis after spinal cord injury
Source: J Tissue Eng Regen Med. 2020 Jan 29;14(3):397–411. doi: 10.1002/term.2996 (PMC7155140; doi:10.1002/term.2996)

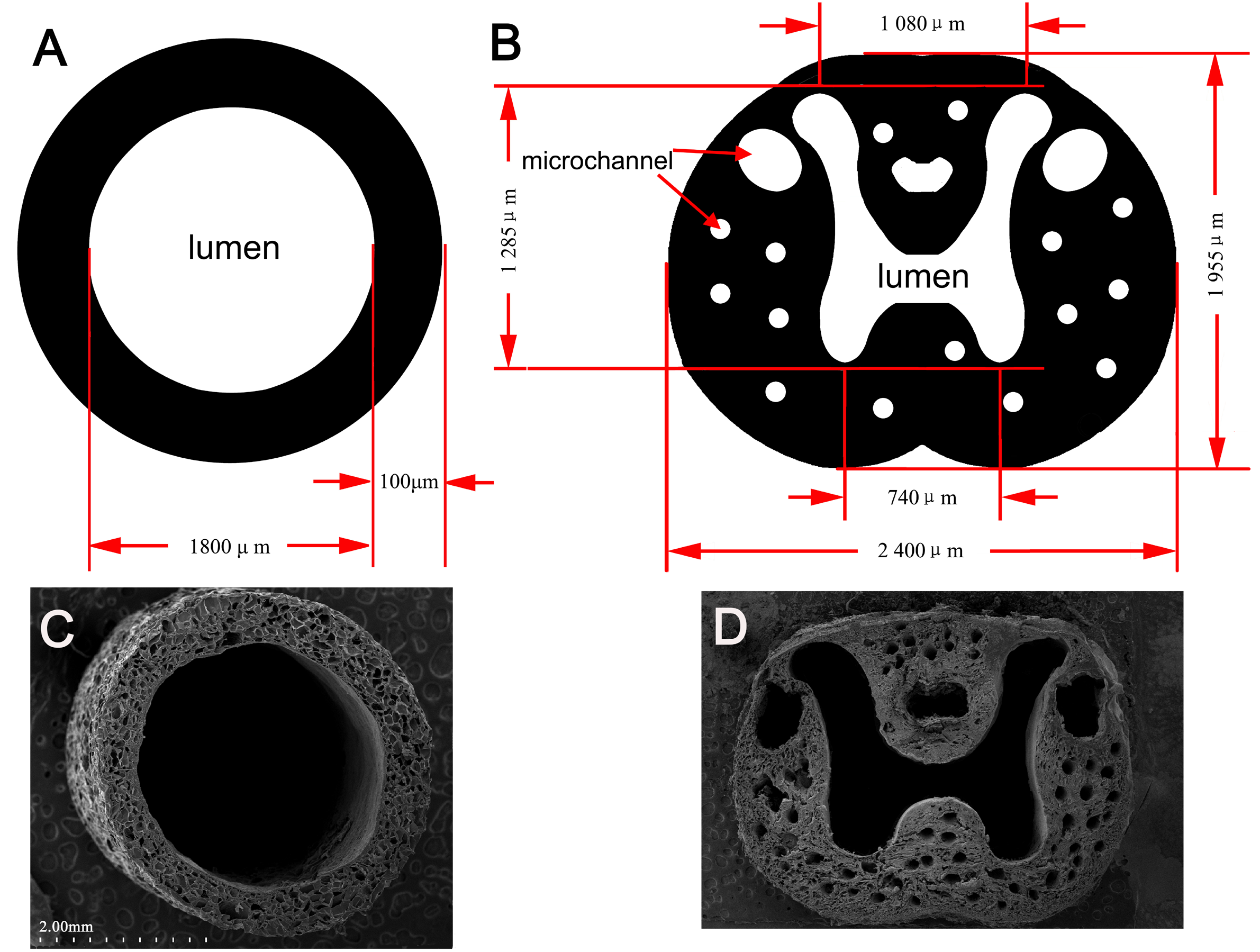

Supplement: Supplementary file 1 — Figure S1. ST and ST/MC for repairing SCI. (A) Schematic diagram of ST design and size, a central empty area and surrounding solid ring represented the circular lumen and wall of the tube, respectively. (B) Schematic diagram of ST/MC design and size, an ‘H’ shape central lumen and surrounding solid tubular wall correspond to the spinal cord grey matter and white matter, respectively; moreover, many microchannels on the tubular wall. (C) ST and (D) ST/MC micrograph on SEM, Scale bar: 2 mm Figure S2. Scaffold with or without BMSCs bridged SCI defect for 12 months. Low power overview of the spinal cord of an animal grafted with ST (A), ST/MC (B), ST + BMSCs (C), or ST/MC + BMSCs (D) bridged to a 5 mm defect at complete transverse thoracic spinal cord for 1 year, regenerated tissue filled the spinal cord defect, integrating the rostral and caudal ends of the spinal cord Figure S3. TEM images of nerve fibers in all groups at 12 months SCI. (A) There were more axon fascicles for myelinated or unmyelinated nerve fibers in the mid‐portion of the regenerated tissues in the ST/MC + BMSCs group. (B) Higher magnifications of area boxed in (A), axons of myelinated nerve fibers wrapped by a thick, uniform and electron‐dense lamellar myelin sheath. (C) There were synapse‐like connections in the regenerated tissues of ST/MC + BMSCs. (D) There were some myelinated or unmyelinated nerve fibers of the regenerated tissues in the ST + BMSCs group, (E) higher magnifications of area boxed in (D). (F) The new capillaries with a well‐established ultrastructure of the regenerated tissues were in the ST + BMSCs and ST/MC + BMSCs groups. (G) and (H) Only a small number of newly formed axons with or without myelin sheath were surrounded by fibroblasts and collagen fibers in the ST and ST/MC groups. (I) There was only glial scar in the SCI group. (J) Bar chart showed significant higher in the number of the myelinated nerve fibers of the ST/MC + BMSCs group. (K) Bar chart showed no significant di [file TERM-14-397-s001.docx]
